# Supplementary material for: Identification of photoperiod-induced specific miRNAs in the adrenal glands of Sunite sheep (Ovis aries)
Source: Front Vet Sci. 2022 Jul 22;9:888207. doi: 10.3389/fvets.2022.888207 (PMC9354845; doi:10.3389/fvets.2022.888207)
Supplement: Supplementary file 1 [file Data_Sheet_1.ZIP › Supplementary file/miRDeep2 file.zip!/miRDeep2 file/Novel_Structure/SPLP42A1/Novel_402.pdf]

Provisional ID	:	NC_019458.2_2202
Score total	:	1.9
Score for star read(s)	:	-1.3
Score for read counts	:	0
Score for mfe	:	1.6
Score for randfold	:	1.6
Score for cons. seed	:	
Total read count	:	146
Mature read count	:	146
Loop read count	:	0
Star read count	:	0

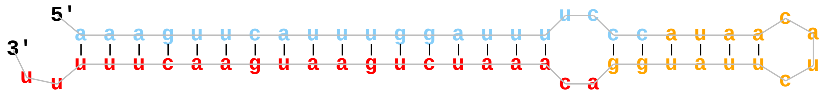

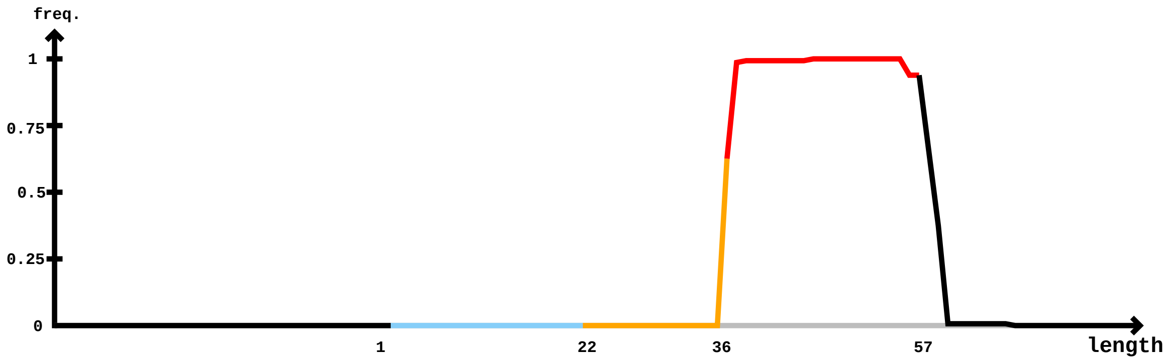

Star Mature

```
5'- cccuaaaccauguaauaucaauugaguuggcaaaaaguucauuuggauuuucccauaacaucuuauggacaaaucugaaugaacuuuuugaccaacccagcagaaucuaga   -3'    exp
    .................((..(((.(((((((((((((((((((((((((..((((((....))))))..)))))))))))))))))))).))))).))).)).......    reads    mm    sample
    ....................................................................aAaaaucugaaugaacuuu........................    9        1     seq
    ....................................................................aAaaaucugaaugaacuuuuu......................    80       1     seq
    ....................................................................acaaaucugaaugaacuuuuug.....................    3        0     seq
    .....................................................................Aaaaucugaaugaacuuuuuga....................    53       1     seq
    ......................................................................aaaucugaaugaacuuuuuga....................    1        0     seq
    .............................................................................aaugaacuuuuugaccaaccU.............    1        1     seq
```
